# Health Tract, No. 254

**Published:** 1866-10

**Authors:** 


From the Boston Watchman and Reflector, by Dr. W. W. Hall, M, D.

HEALTH TRACT, No. 254.

---

### WARNINGS TO MINISTERS & OTHERS.

One of the ablest men of his time, a loved son of New England, gentle as a woman in his manners, but in mind as to culture, and power, and vigor in arguments, a very samson, after preaching in a country church on a cold winter's night, was invited to a neighbor's house until the morning. He retired early, and as usual, was put in the best room, to occupy a most faultlessly clean, soft, white bed. From long disuse it had become damp. He felt its coldness keenly, but not wishing to give trouble, and in the hope of soon becoming warm, he fell asleep, but awoke in the night with a terrible chill and cramp, of which he died in a few hours.

The immediate cause of the death of Lord Bacon, whose renown is world-wide, was the cold and dampness of a spare room; the best room in the house of a friend with whom he stopped for a night on his way to London.

Let parishioners who may chance to read these lines, and who wish to honor a clergyman who may be enjoying their hospitality, with the best things they can offer for his convenience and comfort, have a care to freshly air and warm the bed-clothes of the spare chamber for two or three hours before they are used for the night, especially if the bed has not been occupied for a week or two. If during the evening he has been preaching, give him facilities for being thoroughly warmed before he is sent to his chilly "spare chamber." The clergy of the Christian church are the salt of the earth in a most important sense, for they are the ambassadors of God; hence our interest and duty demand that for their office' sake if for no other, care and consideration should be shown them. No one will be sorry at the judgment for bestowing such attention. The reward will be the same as if it had been done for the Master in His own person, for His words are, "Inasmuch as ye did it unto one of the least of these, ye did it unto Me."

To mothers in Israel another word of caution may be given. Some of the clergy are killed by piecemeal, others in a night, by mistaken kindness. Ye meant it unto good, but the sure result follows for all that, and inevitably. If the minister dines with you and has to preach within a few hours, it is safer and better to provide a very plain meal, so as not to tempt the appetite; otherwise an inefficient or sleepy discourse is almost an inevitable result. A very hearty supper after a long fast or exhausting religious services, endangers life itself. A very able minister of the Lutheran church, and a loved editor of a religious newspaper, was on his way to attend one of the church councils. He left home early in the morning and travelled until noon, but circumstances were such as to make it inconvenient for him to take dinner and before he could reach the intended stopping place it was late in the evening. He was cold, and hungry, and very much exhausted. The family knew all the circumstances, and in their sympathy for him prepared a "splendid supper." He soon felt recuperated, an hour passed pleasantly in conversation, and in due time all retired for the night. The minister did not appear at the breakfast table. On going to his chamber he was found insensible, and in a few hours died of apoplexy. And this was the result of a hearty meal. He had a weak constitution. An empty stomach was overloaded and in that condition he went to sleep, and death was the consequence.

The first meal after a severe effort of either mind or body, especially if the effort has been protracted should be a very simple one, such as light bread, butter, and a cup of hot drink; then in four or five hours a hearty meal may be safely taken, and is necessary.

